# Uncovering perspectives on physical activity in nursing homes: a qualitative exploration of the experiences of healthcare professionals and family caregivers

**DOI:** 10.1186/s12913-024-11711-8

**Published:** 2024-10-11

**Authors:** Stine Øverengen Trollebø, Kristin Taraldsen, Jonas Saur Heiland, Helen Hawley-Hague, Ellen Marie Bardal, Nina Skjaeret-Maroni

**Affiliations:** 1 Faculty of Medicine and Health, Department of Neuromedicine and Movement Science (INB), Norwegian University of Science and Technology (NTNU), Trondheim, N-7491 Norway; 2 Faculty of Health Sciences, Department of Rehabilitation Science and Health Technology, Oslo Metropolitan University (OsloMet), St. Olavs Plass, Postboks 4, Oslo, 0130 Norway; 3 Division of Nursing, Social Work and Midwifery, School of Health Sciences, University of Manchester, Jean McFarlane Building, Oxford Road, Manchester, M13 9PL UK; 4 Clinic of Rehabilitation, St Olavs Hospital, Trondheim University Hospital, Trondheim, Norway

**Keywords:** Nursing home, Physical activity, Neuropsychiatric symptoms, Healthcare professionals, Family caregiver, Older adults

## Abstract

**Background:**

The ageing population has increased the demand for healthcare services. In Norway, community-based long-term care are prioritised, leading to fewer nursing home places. As a result, nursing home residents are now older and have more complex needs. Nearly 92% of nursing home residents are affected by cognitive impairments accompanied by neuropsychiatric symptoms (NPS) that affect their daily activity, physical function, cognition, and behaviour. Traditionally, pharmacological therapy has been the prevailing treatment for NPS. However, emerging evidence suggests that physical activity can serve as an alternative treatment approach. Physical activity has the potential to maintain physical independence and enhance the quality of life (QoL) for the residents. Despite these benefits, institutionalisation in a nursing home often restricts activity levels of residents. This study explores facilitators and barriers to physical activity in nursing homes through the experiences of healthcare professionals and family caregivers. The goal is to enhance our understanding of how to promote and support physical activity for nursing home residents by identifying essential factors for successfully implementing daily physical activity initiatives.

**Methods:**

Seven focus groups were conducted with a total of 31 participants. Participants included healthcare professionals (physiotherapists, nurses, unit- and department managers, assistant occupational therapists, and assistant nurses) and family caregivers of residents at nursing homes. Data were analysed using Braun and Clarke’s reflexive thematic analysis, underpinned by hermeneutic phenomenology.

**Results:**

Three main themes related to facilitators and barriers to physical activity in nursing homes were identified: inconsistency in task prioritisation; need for improved interprofessional collaboration; and need for improved utilisation of external resources. The participants experienced task prioritisation and lack of interdisciplinary collaboration as barriers to physical activity. The involvement of external societal resources was considered as both a facilitator and a necessity for obtaining physical activity in nursing homes.

**Conclusions:**

This study highlights the need for a consensus in task prioritisation, enhanced competence among healthcare professionals, and better interdisciplinary collaboration to facilitate physical activity in nursing homes. Involving external societal resources could be a strategic approach to address barriers and support physical activity initiatives. Future research should focus on developing effective strategies for interdisciplinary collaboration that prioritises and promotes physical activity in nursing homes.

**Supplementary Information:**

The online version contains supplementary material available at 10.1186/s12913-024-11711-8.

## Background

The demand for health- and care services will increase in the coming years as the number of older adults in the population increases [1]. There has been a shift in the financing and operation of healthcare services in Norway, moving from the counties to the municipalities [2–4]. Since then, community-based long-term care has been prioritised, resulting in a reduction in nursing home places [5]. However, despite this reduction, nursing homes still constitutes the largest institutional services in Norway, with approximately 39 000 beds [6].

Norwegian nursing homes are obligated to follow “The national regulation of quality of care” which requires that all residents receive individual and fundamental care, along with meaningful daily activities [7]. This regulation aims to ensure necessary standards for quality of care [7]. However, quality of care in nursing homes is a complex phenomenon, with no consensus on how it should be defined [2]. Consequently, Norwegian municipalities have no mandatory regulations concerning the employee-to-resident ratio, or to the composition of different healthcare professionals within the nursing home teams [8]. Nonetheless, most employees at Norwegian nursing homes possess a formal education, and the staff group typically consist of an interdisciplinary team comprising 51% assistant nurses, 28% nurses, and 2% who are either social educators, occupational therapists, or physiotherapists as well as 19% assistants who do not have any formal healthcare education [9].

Despite a well-educated staff group, the capacity and competence in nursing homes have not kept pace with the increased responsibility for the growing number of patients with complex care needs [10]. As the Norwegian population is getting older, the nursing home population comprises individuals of higher age, and with extensive care needs that require comprehensive support [11, 12]. Almost 92% of Norwegian nursing home residents have cognitive impairments with accompanying symptoms [13]. These symptoms are referred to as neuropsychiatric symptoms (NPS) and include delusions, hallucinations, agitation, dysphoria, anxiety, apathy, irritability, euphoria, disinhibition, aberrant motor behaviour, circadian rhythm disturbance, and appetite abnormalities [14]. NPS pose a significant challenge for healthcare services as they affects the daily activity, physical function, cognition, and behaviour of nursing home residents, and cause distress and a diminished quality of life (QoL) for residents, their family caregivers, and healthcare professionals [11–13, 15, 16]. Treatment of NPS has become challenging as it aggravates the already demanding nature of caring for nursing home residents. Therefore, finding strategies to manage these symptoms are crucial for the well-being of residents, their family caregivers, and healthcare professionals in nursing homes.

Today, pharmacological therapy is the prevailing treatment option for NPS [17, 18]. Nonetheless, medical treatment may lead to several adverse effects such as increased confusion, agitation, sedation, and falls [19]. Physical activity has recently emerged as an alternative treatment approach to improve NPS [17, 20]. Several types of activities lasting from 2 to 5 months, such as multimodal-, endurance-, and strength training has been proven to enhance cognitive function, reduce NPS, and improve QoL among individuals with dementia [20]. Moreover, physical activity has been suggested to positively influence agitation in individuals with dementia [21]. Despite the potential benefits of physical activity, admission to a nursing home is associated with an inactive and passive lifestyle and studies have shown that even ambulatory residents spend up to 94% of their waking hours being inactive [12, 21].

Reduced physical activity can contribute to a decline in physical function, given the well-established dose-response relationship between physical activity and physical function among older adults [22]. A decline in physical function is further associated with a greater need for care, elevated healthcare costs, and a reduced QoL for residents in nursing homes [23, 24]. Research has shown that maintaining the ability to perform the sit-to-stand activity is linked to a slower rate of functional decline, and that this ability is essential as it is closely connected to basic daily activities in nursing homes, such as toileting and dressing [25]. Although nursing home resident face various health challenges, they are generally capable of understanding instructions and participating in activities if they receive sufficient assistance [26–29].

Despite the acknowledged benefits of physical activity, it has been shown that nursing homes provide inadequate levels of physical activity for the residents [2, 30]. A survey conducted in Norway revealed that seven out of ten nurses believe that nursing home residents are not adequately engaged in daily activities [31]. Another study showed that higher staffing levels were not automatically associated with more activities at the nursing home but that physical activity is a complex matter that needs to be investigated [2]. Hence, the aim of this study is to explore the experiences of healthcare professionals and family caregivers regarding facilitators and barriers to physical activity in nursing homes. By gathering insights from both healthcare professionals and family caregivers, we aim to enhance the understanding of how to promote and support physical activity for nursing home residents. Moreover, these experiences may help identify essential factors for successfully implementing initiatives to promote daily physical activity in nursing homes.

## Methods

### Study design and setting

A qualitative design using focus groups was conducted, providing a range of ideas, feelings, and experiences of individuals about a certain topic [32]. Hermeneutic phenomenology informed the qualitative design, as this methodological approach aims to explore the experiences and perspectives of participants. The study took place in a large municipality in Norway that has several nursing homes. Norway serves a public healthcare service where the jurisdiction lies within the municipalities. This means that the municipalities are responsible for providing healthcare services to all individuals residing in the municipality. Therefore, nursing homes may differ across various municipalities, depending on factors such as the size of the municipality, financial resources, political priorities, and demography [33]. In our study context, all included healthcare professionals were located at the nursing homes, except for the physiotherapists who were located externally and delivered a service into the nursing homes based on individual referral.

### Participant recruitment and sample

Purposive sampling was applied to obtain variation in the experiences of healthcare professionals at nursing homes working closely with residents, and family caregivers of the residents. Healthcare professionals were recruited from all nursing homes in the municipality and were considered eligible for inclusion if they worked at a nursing home. No further inclusion or exclusion criteria were applied. Potential participants were contacted via email and provided with information regarding the study. Family caregivers were recruited through participation at a family caregiver meeting organised by the resource centre for dementia in the municipality. No exclusion criteria were applied for this population. All participants who wanted to participate in the study was provided with oral and written information about the study, and all participants signed a consent form prior to the focus groups.

A total of 25 healthcare professionals representing 14 different nursing homes, and six family caregivers with experience from six different nursing homes, participated in this study. All nursing homes were public and owned and run by the municipality. Most participants were females (*n* *= 20)* and ranged in age from 23 to 82 years (see Table 1). Focus groups of healthcare professionals were separated based on profession, which were: physiotherapists, nurses, unit- and department managers, assistant occupational therapists, or assistant nurses. These healthcare professionals were chosen as they are the ones working directly with nursing home residents on a daily basis and represent the front-line staff at nursing homes. The focus group of family caregivers included individuals who were spouses, partners, daughters, and sons-in-law, and were included as research has shown how they provide extensive care towards nursing home residents in Norway [10].

**Table 1:** Characteristics of the participants

	Male/female (*n*)	Age years (range)	Years of working at nursing home/years as family caregiver (range)
Focus group 1: Physiotherapists	3/1 (4)	32–57	2–12
Focus group 2: Physiotherapists	1/4 (5)	23–45	0.25–10
Focus group 3: Nurses	0/4 (4)	24–54	0.4–5
Focus group 4: Unit – or department managers	1/3 (4)	39–56	6–25
Focus group 5: Assistant occupational therapists	0/4 (4)	26–55	1–15
Focus group 6: Assistant nurses	3/1 (4)	34–55	0.25–10
Focus group 7: Family caregivers	3/3 (6)	57–82	0.83–2.5

### Data collection

The research team collaboratively developed an interview guide rooted in hermeneutic phenomenology, comprising open-ended questions tailored to explore facilitators and barriers to physical activity in nursing homes. A pilot interview was conducted by two of the authors, with SØT interviewing JHS prior to data collection, ensuring that the interview guide contained questions that covered the entire area of interest. No revisions were made to the interview guide following the commencement of the study.

Data were collected from June–October 2023. Focus groups with healthcare professionals were conducted separately based on the five different healthcare professions. The focus groups lasted between 65 and 104 min. Six were digitally audio recorded with Marantz pmd661 mkii and one with the encrypted Dictaphone app provided by Nettskjema [34]. For the focus group recorded through Nettskjema, the transcription was initially performed automatically using Whisper Artificial Intelligence (AI) technology. The AI-generated transcript was further manually reviewed by the first author (SØT), and all transcripts were anonymised through verbatim transcription.

### Data analysis

A reflexive thematic analysis was conducted using Braun and Clarke’s six-phase process, as it allows for a nuanced exploration of subjective experiences and interpretations within the data [35]. The entire analysis was performed using a hermeneutic phenomenological lens, allowing the authors to interpret and reflect around deeper meanings attached to the data [36]. In the first phase, the first author (SØT) read and re-read the transcripts to familiarise with the data. Several transcripts were additionally read by three of the authors (KT, NSM, and JSH), and all noted initial thoughts, impressions, and questions about the data. In the second phase, the data were coded using the software NVivo 14 to organise and manage the data. Codes were assigned to segments of data that captured their meaning or significance using inductive coding, meaning that no theoretical perspective guided the coding process. Additionally, a latent coding approach was applied to assign underlying assumptions, meanings, and ideas to the data. The third phase consisted of collating the codes into broader categories that made up the preliminary themes which in phase four were revised to check their coherence with the data and the research objective. As barriers and facilitators often describe different aspects of the same phenomenon, they were analysed together. However, it was noted whether the data explained a barrier or facilitator. To increase rigor, the first and last authors (SØT and NSM) independently performed this phase before the authors met to discuss and revise the themes and to reach a consensus. This revision consisted of merging, splitting, and discarding some of the preliminary themes. The authors iteratively navigated through the different phases to refine and deepen their understanding of the data. In the fifth phase, themes were named and defined with informative labels describing the scope of the data. Furthermore, the authors ensured that the themes had consistent narratives and that they represented the perspectives of the participants. At every stage, the themes were discussed in the team and the viewpoints of the authors and their potential influence, considered. In the final phase, the analysis was written by presenting the themes in a logical order, illustrating them with quotes from the participants, and discussing their implications for policy and practice. All quotations were checked in English by a native speaker (HHH). To answer the research questions, the focus groups of healthcare professionals and family caregivers were presented together.

### Analytic lens

The results will be discussed in relation to the Canadian Interprofessional Health Collaborative Framework (CIHC), containing competency domains that centres around the development and integration of attitudes, behaviours, values, and judgments considered essential for good collaborative practice [37]. The CIHC framework was chosen as it provides a structured approach to evaluating the competency domains that are crucial for fostering effective interprofessional collaboration in healthcare settings.

### Ethical considerations

Ethical approval was obtained from The Norwegian Agency for Shared Services in Education and Research (SIKT, ref. nr. 866138) prior to study commencement. All participants received written and oral information about the study as well as their right to withdraw from the study at any time without giving a reason. Informed, voluntary written consent was obtained before the focus groups. It is also important to acknowledge that the researcher was responsible for respecting the voices of the study participants, ensuring that interpretations remained unbiased by the researcher’s own perspectives and potential preconceptions. Given that the first author (SØT) did not share a background as a healthcare professional, it is reasonable to assume that the interactions were free from any personal bias or familiarity. In the process of writing, AI was used for language checking by an internal language model tool to ensure that the data remained within the organisation and to minimise the risk of open sharing.

## Results

Based on the perspectives of healthcare professionals working with nursing home residents and the family caregivers, we identified three main themes related to facilitators and barriers for physical activity in nursing homes: (1) inconsistency in task prioritisation, (2) need for improved interprofessional collaboration, and (3) need for improved utilisation of external resources. Table 2 shows an overview of the main- and sub themes.

**Table 2:** Themes identified in the analysis

Main Themes	Sub Themes
Inconsistency in task prioritisation	Definition of primary needs
	Need for increased competence
Need for improved interprofessional collaboration	Clarification of roles and responsibilities
	Improved interprofessional insight
Need for improved utilisation of external resources	Limited resources at the nursing home
	The ambiguous role of family caregivers

### Inconsistency in task prioritisation

Our healthcare professionals and family caregivers experienced challenges regarding inconsistency in task prioritisation, identifying this as a barrier to physical activity in nursing homes. Many healthcare professionals expressed the feeling of being under constant time pressure at work, finding themselves rushing from one task to another to manage urgent needs and demands. This time pressure was further attributed to staff shortages at the nursing home, and many healthcare professionals stated how this resulted in established norms and routines prioritising the primary needs of the residents. One nurse explained this prioritisation, stating: *“What needs to be done*,* the basics*,* is prioritised*,* and there’s barely time to do these tasks. Therefore*,* physical activity comes quite far down the priority list.”*, P4. However, many healthcare professionals had different views regarding what the primary needs of nursing home residents were. While many healthcare professionals considered administration of medication, feeding, hygiene, and toileting as the main primary needs, others advocated for prioritising physical activity as an equally important need. One assistant nurse said: *“I kind of believe that physical activity should be very important*,* it’s like it is with food*,* care and other needs*,* physical activity is very important too.”*, P1. This view on the prioritisation of physical activity was supported by several family caregivers, emphasizing the importance of physical activity on the mental health of the residents. One family caregiver stated: *“But I think that physical health should be raised quite high*,* because it has to do with the mental health as well. So*,* I think that if we are going to prioritise*,* then physical health must be prioritised quite high.”*, P6.

Furthermore, there was an agreement amongst several healthcare professionals and family caregivers that physical activity receives insufficient attention within the nursing home setting. This lack of attention was partly explained by the fact that no single healthcare profession bears sole responsibility for encouraging physical activity, in addition to limited awareness and knowledge among the staff. In particular, one unit manager explained how many healthcare professionals lack knowledge on the benefits that physical activity can offer to the residents, saying: *“I also think that it’s the knowledge of the staff*,* the thing about how little activity is actually needed to improve physical health*,* I think it’s too low.”*, P3. Following this statement, suggestions were made on how physiotherapists could contribute to increasing the competence of respective healthcare professionals at the nursing home around physical activity. One unit manager specified how they regularly were provided with competence development opportunities, but found it challenging to effectively pass on newly acquired knowledge to the other rest of the staff at the nursing home due to time constraints:

*“We are not able to share it with all the 70 healthcare professionals*,* we are far too poorly covered or staffed*,* we have staff meetings*,* and we have other networks*,* and we have user meetings and meetings with relatives in addition to everything we have to do during the day*,* so then we do not have the time to share.”*, P3.

### Need for improved interprofessional collaboration

Many healthcare professionals highlighted that the complexity of caregiving at the nursing home made it challenging to allocate tasks according to profession. This inadequate division of responsibilities were explained through staff stating that they took on multiple roles, going beyond the traditional tasks designated to their healthcare profession. One nurse shared the feeling of being overwhelmed by the range of tasks they have to perform, and suggested how a better interprofessional collaboration could be beneficial to their workload, saying: *“We do everything that assistant nurses do*,* plus the rest. So*,* it might have been an idea if we didn’t have to do all the tasks. After all*,* we fold clothes and fill the dishwasher and do all these tasks. Clean the tables and…”*, P4. Several healthcare professionals shared the view of undefined division of responsibilities as problematic and attributed it to poor interprofessional understanding. It was explained how different healthcare professionals lacked recognition of each other’s contributions to the staff group and towards the nursing home. Some healthcare professionals further stated how this led to reduced utilisation of the diverse perspectives and capabilities within the different healthcare professions, and two assistant occupational therapists expressed how this fostered a negative culture characterised by diminished respect for each other’s contributions to the interdisciplinary team: “*I’ve heard many times that: “You’re just an assistant occupational therapist”*,* which is a bit… “You’re just an assistant occupational therapist.”*, P1. *“And there are never substitutes for us if we are sick. Or on sick leave. Because there is no need for us…”*, P2.

Furthermore, beyond the absence of insight into the contributions of other healthcare professionals, one physiotherapist acknowledged little clarity regarding their own role and responsibilities within the nursing home:

*“Suddenly a referral came from the nursing home*,* asking for the responsible person. Then I asked*,* “Who is the responsible person?”*,* and everyone just*,* “I don’t know”. Then we checked the list*,* and it was me. When I asked the person next door what I should do*,* I got the answer: “I don’t know”.”*, P4.

This statement suggests poor communication and uncertainties around where to contribute to the nursing home. As a result to this problem, another physiotherapist suggested that actively participating in the nursing home environment, including physical presence, could lead to a deeper understanding of where to contribute, as well as an increased sense of responsibility. It was further emphasised that physical presence could benefit the residents by observing their potential, and one physiotherapist said: *“Just having a meeting point*,* being present*,* being able to talk about patients together to see what the potential is.”*, P5. The benefit of physical presence and increased involvement of physiotherapists in the staff group at the nursing home was supported by one of the department managers, who stated: *“Having the physiotherapists as a part of the staff*,* one physiotherapist for each nursing home that could help with teaching and guidance in our everyday life*,* that would have been amazing.”*, P1.

### Need for improved utilisation of external resources

Alongside the pressured situation experienced by staff related to staff shortages and recruitment challenges within nursing homes, many healthcare professionals and family caregivers expressed the need for improved utilisation of external resources within society. One family caregiver implied how nursing homes are not able to manage without help, stating: *“Volunteers and relatives need to be involved to a much greater extent. I don’t think nursing homes can manage without help”.* P5. This view was supported by one department manager, who pointed out an unexploited potential among different groups from society:

*“We have many people who are in job training or on disability that wants to work and that have a work capacity of 20%*,* but we can’t figure out where to use them”. Maybe I’m being strict*,* but what if we say that they need to go to a nursing home*,* and just walk with the residents down the corridors once or twice. I believe we have much more potential to utilize.”*, P3.

Although many participants highlighted the necessity for increased engagement and participation from society, the ambiguous role of family caregivers appeared as a topic with divergent opinions. While some healthcare professionals highlighted the necessity for increased engagement and participation from family caregivers, especially in terms of carrying out physical activity initiatives with the residents, others expressed that the primary responsibility for all care should lie with the healthcare professionals at the nursing home. One assistant nurse expressed a wish for contribution from family caregivers, saying: *“Many times*,* I have wished that family caregivers could contribute with taking their mother out for a little walk or something like that.”*, P2. On the other hand, several family caregivers endorsed not having any designated duties at the nursing home, explaining inclusion of family caregivers as an unstable and unreliable resource, potentially leading to greater differences among the residents: *“I don’t think that family caregivers should have any designated duty towards the nursing home. You should visit your husband when you want to*,* not when you have to.”*, P5. However, one family caregiver expressed a desire to be more involved but explained an uncertainty about where and how to contribute. The family caregiver further explained that there is no clear description of their role, leading to uncertainties around what the nursing homes expect from them:

*“There must be proposals from them because I have heard “Please do something”*,* but we don’t know what to do? I almost feel like the fifth wheel on the wagon when I’m there. It must be signalled from the nursing home*,* where they want us to contribute.”* P6.

## Discussion

This study aimed to investigate the experiences and perspectives of healthcare professionals and family caregivers concerning facilitators and barriers to physical activity in nursing homes, aiming to deepen our understanding of how to effectively encourage and support physical activity in these environments. A main finding was that inconsistency in task prioritisation acted as a barrier to physical activity. Our participants explained this prioritisation to be a result of inconsistency around what defines a primary need, as well as lack of competence among the staff. Furthermore, a lack of collaboration within the interdisciplinary staff group was highlighted as a barrier to physical activity. The lack of collaboration was explained by poor understanding of each other’s contributions to the collective effort, compounded by time and resource constraints. As a solution to the time and resource challenges, it was emphasised by both healthcare professionals and family caregivers that there is a need for greater involvement of external societal resources.

Healthcare professionals in our study discussed how inconsistency in task prioritisation acted as a barrier to physical activity in nursing homes, explaining how they frequently postponed or neglected physical activity due to competing demands. This practice, known as rationing, involves making reasoned decisions for limited care [38]. Rationing is widely observed across several healthcare settings in Norway and is commonly ascribed to multiple workplace factors, including stressful situations, support from colleagues, overall job satisfaction and lack of resources [3, 39]. Moreover, rationing is connected to how staff prioritise tasks, often concentrating on urgent duties and neglecting less mediate responsibilities [40]. Research has demonstrated how nursing home staff in Norway prioritise basic physical needs like feeding, bathing, and giving medicines, and giving less attention to socializing and comforting residents [41]. Our results revealed conflicting opinions on which tasks to prioritise. Several healthcare professionals argued that physical activity is not a primary need, whereas others, including both healthcare professionals and family caregivers, stated that physical activity should be prioritised, especially due to its benefits in supporting mental and physical health. These perspectives were not inherently tied to specific healthcare professions but reflected rather individual perceptions and beliefs about the importance of physical activity. This suggests a lack of consensus on its importance for the residents, highlighting the need for clear guidelines on what constitutes physical activity in nursing homes. Considering the challenges of managing NPS among nursing home residents, leveraging the benefits of physical activity could potentially enhance the well-being of everyone involved.

Our results further showed that neglecting physical activity may be a result of lacking competence among the staff, and it was suggested that frequent staff turnover, staff shortages, and insufficient time for knowledge transfer were reasons for this. These experiences are consistent with previous studies which revealed that nursing home staff often lack sufficient competence, and that Norwegian municipalities fail to provide necessary competence development consistent with the complex care of older adults [42–44]. Insufficient staff competence may further work as a mediating factor influencing the ability to integrate physical activity initiatives in nursing homes, neglecting the minimal yet effective movements. Previous research has demonstrated that the sit-to-stand activity benefits the mobility and function for residents and that this activity is crucial for participation in daily activities within the nursing home [25]. Another study demonstrated that maintaining consistent participation in this activity is challenging for nursing home residents, and that feedback and monitoring by healthcare professionals may increase adherence [45]. Sit-to-stand activities can easily be integrated in everyday life with the resources that already exists at the nursing homes and should therefore be prioritised given the benefits it provides to the residents.

Furthermore, many healthcare professionals in our study expressed feeling overwhelmed by numerous tasks and stated that no specific healthcare profession is responsible for ensuring physical activity for the residents. It was suggested that the physiotherapist could take responsibility for physical activity by increasing their physical presence in nursing homes. Moreover, physiotherapists could enhance the competence of respective healthcare professionals and serve as an available resource and a consistent reminder to prioritise physical activity for residents. Previous research has highlighted that physiotherapist play an important role in integrating physical activity into the everyday life of nursing home residents [46]. However, inconsistent organisational and personal factors within these settings affect their involvement [46]. Given the increasing complexity and frailty of nursing home residents, we advocate for greater inclusion of physiotherapists and clearer role distinctions among the different healthcare professionals, to ensure that physical activity is appropriately prioritised.

Clear role descriptions and explicitly communicated responsibilities within an interdisciplinary team, is described by the CIHC framework as a prerequisite for effective teamwork [37]. Effectively delegating tasks and responsibilities can contribute to a sense of ownership and enhance interdisciplinary insight. Nevertheless, many healthcare professionals in our study expressed a reality consisting of little insight into each other’s contribution to the nursing home and explained how each healthcare profession worked as separate groups. This finding corresponds to a study conducted by Tsakitzidis and colleagues (2017), which revealed that different healthcare professions in nursing homes work separately from each other and lack collaboration towards a common objective [47]. The CIHC framework proposes how working towards a common goal may establish a collaborative foundation that promotes respect for diversity within the staff, potentially fostering a culture of mutual appreciation and shared responsibility [37]. Such comprehension can further contribute to a seamless coordination of tasks, potentially preventing redundancies and gaps in care.

Nonetheless, Norwegian nursing home services presents a reality based on staff shortages, and both healthcare professionals and family caregivers expressed a need for greater inclusion of external societal resources such as volunteers. The leaders specifically pointed out an unexploited potential among people outside the nursing home to conduct physical activity with the residents. Research has shown that involving volunteers in nursing homes provides positive outcomes for residents, notably through companionship, which boosts mood and engagement [48]. One study highlighted the benefit of volunteer-led activities that challenge the resident’s physical, emotional and social capacities, such as manual tasks, memory projects, and games [49]. Additionally, given the benefits of physical activity in reducing NPS [20], there is potential for engaging volunteers to facilitate these activities. This collaborative strategy not only improves the QoL for the resident but also improves the QoL for healthcare professionals, family caregivers, and volunteers [49].

Along with inclusion of external societal resources, several healthcare professionals proposed greater inclusion of family caregivers in nursing homes. It was proposed how family caregivers could serve as a resource and participate in tasks they frequently omit, such as physical activity. This was, however, an engaging topic with divergent opinions. On the contrary, family caregivers mainly expressed challenges associated with their involvement in the nursing home and raised concerns about a future nursing home service that heavily relies on their involvement. They explicitly feared that such reliance could lead to greater differences in provided care to the residents, particularly concerning physical activity initiatives. Previous research supports these concerns, stating that the role of family caregivers should not extend beyond desired involvement and that the unpredictability of these resources can strain the infrastructure within the nursing home [50]. Given that involvement family caregivers can vary based on availability, geographic proximity, and the nature of the relationship, it may lead to greater differences between residents [50, 51].

Moreover, several of the family caregivers expressed uncertainties regarding where and how to contribute to the nursing home. This illustrates the need for improved clarification and communication between the nursing home and family caregivers, which is reflected in the principle of patient-centred care in the CIHC framework [37]. This domain underscores the importance of clear role clarification and effective communication toward providing care that is respectful, individually tailored, and inclusive for all involved parts. Furthermore, the principle addresses the importance of sharing information with family caregivers in a respectful manner that is understandable as well enhancing participation in decision-making. Incorporating the principle of patient-centred care may be a step toward creating an environment where the well-being of residents stands at the forefront of every decision and action.

A strength of this study was that it involved participants from different healthcare professions representing the front-line staff working in nursing homes, as well as family caregivers. This ensures that diverse perspectives on physical activity are captured. Furthermore, the rigorous analytical process with separate analyses followed by team discussions, enhances the trustworthiness of the results. However, the study did not involve nursing home residents in the focus groups, due to practical and ethical challenges. This may lead to important perspectives being overlooked, undermining the representativeness of the findings. Moreover, the study population exhibited a gender imbalance with a predominance of females, potentially affecting the generalisability of the findings to the broader population. However, this gender imbalance reflects the landscape of healthcare professionals, where women constitute a significant majority [52]. Finally, we included a single municipality in Norway that may restrict the broader applicability to other regions and contexts.

## Conclusion

This study explored the perspectives of healthcare professionals and family caregivers regarding facilitators and barriers to physical activity in nursing homes. Our qualitative investigation revealed challenges related to prioritising physical activity, ensuring sufficient competence, and promoting effective interdisciplinary collaboration as barriers to physical activity in nursing homes. Moreover, greater involvement of external societal resources to conduct physical activity initiatives with the residents was described as both a facilitator and an unexploited solution. These findings underscore the urgency for collaborative efforts and targeted interventions to enhance physical activity in nursing homes. Future research should therefore aim to identify effective strategies for interdisciplinary collaboration that prioritises and promotes physical activity in nursing homes.

## Supplementary Information

Supplementary Material 1.

## Data Availability

The datasets generated and analysed during the current study are not publicly available due to privacy concerns that could compromise the confidentiality of participants but are available from the corresponding author on reasonable request.

## Abbreviations

NPS: Neuropsychiatric symptoms
QOL: Quality of Life
