# Supplementary material for: Uncovering perspectives on physical activity in nursing homes: a qualitative exploration of the experiences of healthcare professionals and family caregivers
Source: BMC Health Serv Res. 2024 Oct 11;24:1222. doi: 10.1186/s12913-024-11711-8 (PMC11470674; doi:10.1186/s12913-024-11711-8)
Supplement: Supplementary file 1 — Supplementary Material 1. [file 12913_2024_11711_MOESM1_ESM.docx]

**Physical activity in nursing homes – Focus groups of healthcare professionals and family caregivers**

Interview guide

**Research question:**

(How do healthcare professionals and family caregivers experience facilitators, needs, and barriers to physical activity in nursing homes?)

**Facilitators:**

1. What do you think is good about today's nursing home service?
2. What opportunities do you see for physical activity among nursing home residents?

- Design of the nursing home
- Care
- Meals

1. How aware are the staff of physical activity?

- (Their own activity)
- Activity among the residents

**Needs:**

1. How do you perceive the residents' need for physical activity?

- How does this correspond to the residents' actual activity level?

1. How do you work to engage the residents in the nursing home?

- Who or what influences your ability to engage the residents?

1. If an initiative to increase physical activity is to be implemented at the nursing home, what needs to be done to make this possible?

- Has this been done before?
- How did it go?
- What role does a staff member play in a successful implementation process?

1. Any other aspects you wish to highlight that you believe may be important for implementing increased physical activity in nursing homes?

**Barriers:**

1. What challenges do you experience with today's nursing home services?
2. What challenges do you see for physical activity among nursing home residents?
3. What do you think should be improved with today's nursing home services?
4. What are your thoughts on the level of competence regarding physical activity among the staff?

**Finishing up**

1. Of all we have discussed today, what would you highlight as most important?
2. Is there anything else you would like to add that we have not talked about today?
3. Do you have any questions for me?
